# Rethinking Medical Education Through Connection: A Narrative Review of Cultivating Collaborative Intelligence for Sustainable Healthcare

**DOI:** 10.7759/cureus.89772

**Published:** 2025-08-11

**Authors:** Nobuyasu Komasawa, Masanao Yokohira

**Affiliations:** 1 Community Medicine Education Promotion Office, Faculty of Medicine, Kagawa University, Miki-cho, JPN; 2 Department of Medical Education, Kagawa University, Miki-cho, JPN

**Keywords:** collaborative intelligence, connection-centered education, interprofessional education, medical education reform, sustainable healthcare

## Abstract

Modern healthcare faces unprecedented complexity, driven by aging populations, chronic diseases, global health threats, and widening health disparities. These challenges are deeply embedded in social, institutional, and environmental systems, demanding a shift from isolated clinical practice to dynamic, multi-layered collaboration. Yet, current medical education, both in Japan and globally, often underprepares learners for the relational, interprofessional, and systemic demands of real-world healthcare. This narrative review explores how medical education must be reimagined to cultivate "collaborative intelligence," the ability to engage across disciplinary, generational, institutional, and community boundaries. Drawing on educational theories such as Vygotsky’s social constructivism, we advocate for a connection-centered, practice-oriented curriculum that embeds relational learning across all stages of training. Through redesigned clinical clerkships, interprofessional simulations, community-based rotations, and structured mentorship, students can develop not only communication and teamwork skills but also the ethical reasoning and adaptive leadership required in complex care environments. We also examine structural barriers, including institutional silos, hierarchical cultures, and outdated assessment systems, that continue to impede meaningful collaboration. We argue that embedding connection into the core of medical education is both a pedagogical necessity and a moral imperative. Ultimately, the goal is not only to adapt to the complexity of healthcare systems but also to empower future professionals to transform them toward resilience, equity, and human-centered care. In doing so, medical education can become a catalyst for sustainable health systems and inclusive communities.

## Introduction and background

Modern healthcare is facing unprecedented complexity [1]. Aging populations, chronic diseases, emerging infections, and widening health disparities are straining the ability of health systems to provide equitable, effective, and sustainable care [2]. These are not merely clinical challenges; they are deeply rooted in social, environmental, and institutional structures such as unequal access to services, demographic shifts, and under-resourced communities. Healthcare should therefore be understood not only as a technical domain but also as a fundamentally social endeavor grounded in trust, communication, and collaboration.

In this context, the global pursuit of sustainable development, emphasizing health equity, inclusion, and environmental responsibility, has gained critical urgency [3]. Healthcare lies at the intersection of these priorities: it not only treats illness but also actively shapes the social and structural conditions that allow individuals and communities to thrive [4]. This expanded role makes collaboration essential. No single profession or institution can meet today’s challenges alone. Sustainable care depends on multi-layered cooperation that spans professional, institutional, generational, and community boundaries.

Such collaboration includes teamwork among physicians, nurses, pharmacists, and allied health professionals; learning across roles and generations within departments; partnerships with sectors such as education, welfare, and the environment; and meaningful engagement with communities and civil society [5]. These interconnected relationships form the backbone of resilient health systems. Yet traditional medical education often underemphasizes the competencies needed to build and sustain them.

Historically, medical training has prioritized individual knowledge acquisition and technical proficiency [6]. To meet today’s demands, this model must evolve. Education must intentionally foster learners' ability to connect across roles, institutions, and communities. Communication training, for example, should extend beyond doctor-patient interactions to include interprofessional dialogue, team-based decisions, and community collaboration.

This transformation requires learning environments that emphasize real-world engagement, shared reflection, and collaborative problem-solving. When students learn through interaction, active participation, and mutual support, they not only acquire knowledge but also develop the relational skills and values necessary to thrive in complex healthcare systems [7]. We approach this question through the lens of social constructivism, a theory that sees knowledge as shaped by social interaction and cultural context.

This narrative review explores how medical education can be redesigned to cultivate such relational competencies. Given its theoretical focus, the review does not follow a systematic methodology. However, relevant literature was selected based on purposive sampling of peer-reviewed sources, policy documents, and key conceptual works mainly from 2016 to 2025, identified through expert knowledge and targeted database searches (e.g., PubMed). This approach aims to synthesize diverse perspectives while maintaining conceptual depth and clarity. We argue for a practice-oriented, system-aware, and sustainability-conscious approach, one that prepares future professionals not just to deliver care, but to work across boundaries and help build a more inclusive, resilient, and collaborative healthcare future.

## Review

Rethinking medical systems in crisis: why collaboration across generations and sectors matters

Healthcare is both a reflection and a driver of society’s sustainability. In today’s world, it is no longer sufficient to treat disease in isolation; healthcare professionals must also build bridges between individuals, professions, institutions, and generations to support the collective health of society [8]. The greatest threat lies not only in pandemics or disasters themselves but in the failure to connect. In this pursuit, the most essential quality of healthcare professionals is not only technical competence but the capacity to co-create, adapt, and collaborate across boundaries to shape a healthier, more inclusive future.

This requires a fundamental re-envisioning of medical education. Future healthcare professionals must be equipped not only with clinical skills but also with the relational, ethical, and systemic competencies to navigate complex interdependencies [9]. They must be trained as agents of sustainable healthcare, capable of engaging across disciplinary, generational, institutional, and community lines. This demands curricula that integrate interprofessional education (IPE), community engagement, global health, and sustainability science, reflecting the complexity of real-world healthcare [10].

In recent years, the global call for sustainable development has gained unprecedented urgency [11]. Addressing interconnected challenges such as poverty, health inequities, education gaps, and environmental degradation requires integrated efforts across all sectors. Healthcare stands at a crucial intersection, not only responding to illness but actively shaping the conditions that enable people and communities to thrive. Through treatment, prevention, and daily care, healthcare contributes directly to building a fair, inclusive, and resilient society.

At the heart of this contribution lies collaboration. In increasingly specialized and fragmented health systems, long-term sustainability depends on multi-layered cooperation: among healthcare professionals (physicians, nurses, pharmacists, social workers, rehabilitation specialists), across departments and generations, between hospitals and community services, and in partnerships that link healthcare to welfare, education, and civil society. These layers of collaboration are deeply interconnected; fragility in one area weakens the whole.

The COVID-19 pandemic exposed the consequences of failing to build such integrative structures [12]. In many countries, delayed coordination between public health authorities, hospitals, and primary care led to confusion and preventable harm [13]. In Japan, insufficient collaboration between hospitals and long-term care facilities contributed to avoidable deaths among the elderly, as care homes lacked infection control support and timely hospital transfers [14]. Elsewhere, lack of coordination between health and education sectors exacerbated youth mental health crises, and inconsistent communication between national and local authorities undermined public trust.

These outcomes were not simply clinical failures; they were systemic and relational. Truly sustainable healthcare requires robust, resilient networks that span sectors, disciplines, and generations. Whether through interdisciplinary collaboration across health, education, and environment, or partnerships among governments, universities, and communities, such connections are not optional. They are the foundation of an inclusive and effective response to both chronic challenges and acute crises.

Overcoming them requires a cultural and educational shift. We must understand collaboration not merely as a practical strategy but as a deeply human endeavor, a continuous process of negotiation, mutual learning, and connection. Yet building and sustaining such partnerships remains difficult [15]. Structural divisions, professional hierarchies, and communication gaps persist. Only by embedding this ethos into the core of healthcare can we move toward a more sustainable, equitable, and connected future.

Beyond IPE: rethinking connection in healthcare education

Despite growing awareness of the importance of collaboration in modern healthcare, current IPE frameworks remain insufficient [16,17]. Rather than simply expanding IPE, what is urgently needed is a transformation toward multi-layered, connection-oriented education, one that prepares learners to build meaningful, multi-dimensional relationships across professions, sectors, systems, and generations. Connection-centered education goes beyond traditional IPE by cultivating the collaborative intelligence essential for navigating complex, interconnected healthcare systems [18]. Existing medical education structures, both in Japan and globally, continue to fall short in cultivating these essential relational competencies [19,20].

Curricula in many countries, regardless of income level or system maturity, still emphasize individual knowledge acquisition, technical proficiency, and biomedical reasoning [21]. This focus on individualism is reinforced by competitive admissions, exam-driven assessments, and disciplinary silos, limiting students’ capacity to engage with the complexity of real-world collaboration. In Japan, the 2017 revision of the Model Core Curriculum introduced IPE and community-based learning, but implementation has often been superficial [22]. IPE tends to be limited to brief simulations or classroom sessions, lacking continuity or genuine interprofessional engagement. Medical students rarely experience sustained interaction with nurses, therapists, pharmacists, or care workers in egalitarian team contexts. Similarly, community-based training is often centered around patient encounters, with limited engagement with public health authorities, social workers, or non-clinical actors [23].

These gaps leave students ill-prepared to understand how health intersects with social systems and policy structures. Understanding these intersections is essential for developing professionals who can navigate complexity, address structural determinants of health, and contribute to collaborative, system-level change.

Globally, similar challenges persist. Even in countries like the United States, Canada, and parts of Europe where IPE is formally endorsed, integration is hindered by institutional silos, misaligned schedules, insufficient faculty training, and weak evaluation frameworks [24]. In low- and middle-income countries, the situation is exacerbated by resource constraints, further restricting opportunities for meaningful collaboration [25]. What is striking across all contexts is a persistent neglect of "connection" at multiple levels, not just between professional disciplines, but across generations, sectors, and communities. Medical students are seldom taught how to engage in collaborative decision-making, navigate intergroup dynamics, or build trust with non-clinical stakeholders such as educators, caregivers, or local government officials [26]. Opportunities for intergenerational learning, such as structured mentorship or dialogic exchanges between junior and senior professionals, are rarely prioritized, despite their proven benefits in fostering reflective and adaptive thinking [27].

This disconnect is further reinforced by outdated assessment systems that prioritize technical accuracy and individual performance over collaborative reasoning, communication, and reflective practice [28]. Relational and systemic competencies, when assessed at all, are often reduced to secondary "soft skills" rather than being treated as foundational to quality care, despite their explicit inclusion in core educational frameworks such as the WFME Global Standards for Quality Improvement [29].

At the root of this issue lies a deeper systemic inertia. Achieving truly connection-centered education will require more than adding IPE modules or revising course content. It demands an institutional commitment to relational pedagogy, faculty development, structural support for interprofessional engagement, and a cultural reorientation toward collective intelligence. Without such transformation, medical education risks remaining fragmented and outdated, unable to prepare future professionals to meet the relational and systemic demands of contemporary healthcare.

Embedding connection in clinical training: toward a relationally competent healthcare workforce

To move from theory to meaningful transformation, clinical training, particularly in the form of diagnostic and clinical clerkships, must be restructured as a central platform for cultivating relational competence [30]. These immersive learning opportunities are uniquely positioned to bridge the gap between educational ideals and healthcare realities. Yet in practice, they often fall short of their potential. Medical students may find themselves relegated to the margins of the care team, observing rather than participating, with limited interaction beyond their supervising physician [31]. This siloed model not only stifles engagement, but also undermines the development of collaborative intelligence, which is essential for the practice of modern, sustainable, and inclusive healthcare [32].

**Figure 1:**
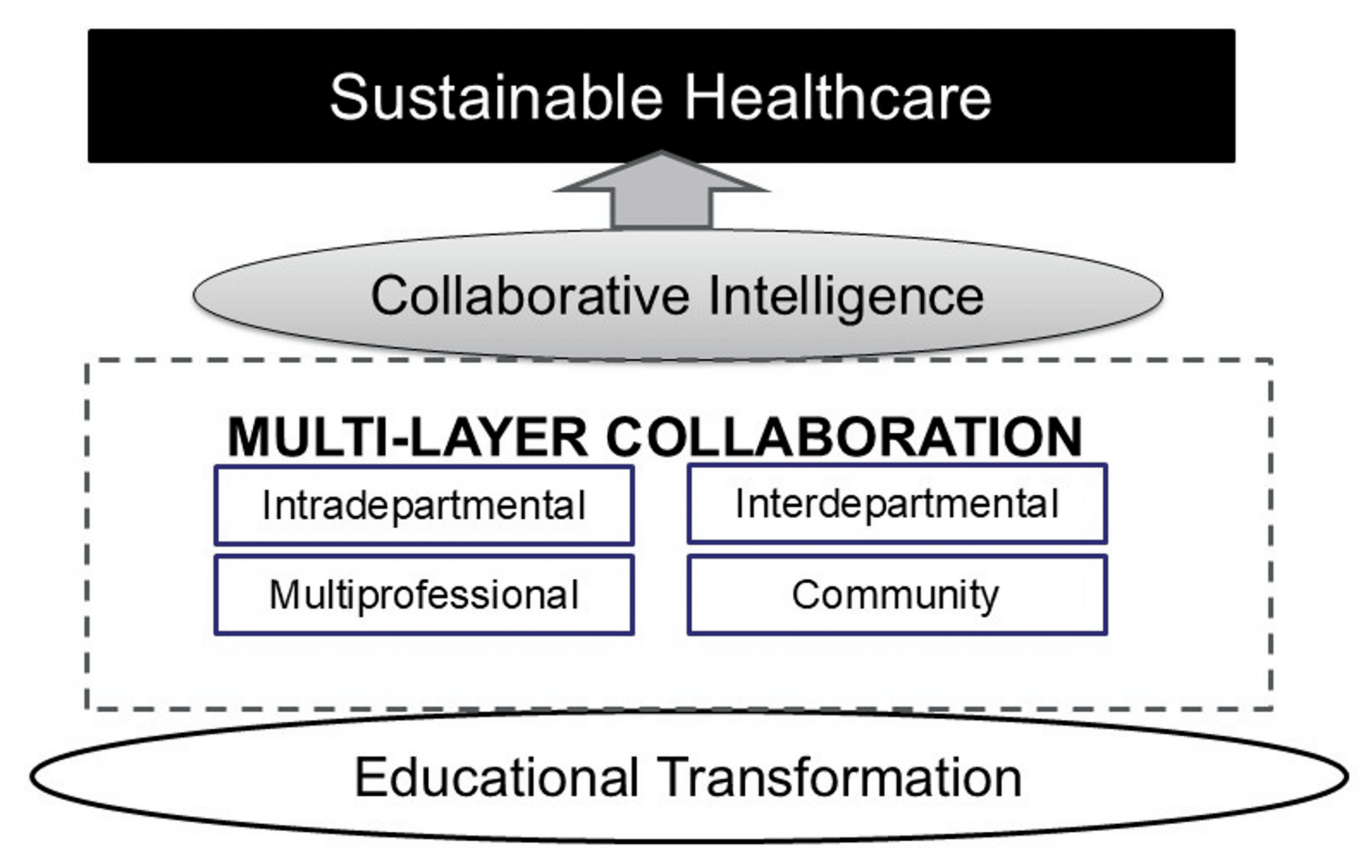
The multilayered collaboration framework for sustainable healthcare.

In contrast, a connection-centered redesign of clinical clerkships would embed students as active contributors within interprofessional care teams [33]. Beyond receiving instructions from senior physicians, students would engage in dialogue and shared decision-making with residents, nurses, rehabilitation therapists, pharmacists, social workers, care coordinators, and administrative staff [34]. Each profession brings a unique lens to patient care, and deliberate exposure to these perspectives enables students to move beyond narrow disciplinary thinking. Structured opportunities for interprofessional case discussions, collaborative rounds, and role-shadowing experiences should be incorporated systematically throughout the curriculum [35]. Such strategies not only build respect and understanding across professions but also reinforce that high-quality care is inherently team-based, as illustrated in Figure 1.

Table 1 illustrates a structured framework for connection-centered medical education, highlighting the central role of clinical clerkship in the latter half of the curriculum. Importantly, these experiences must also be socially and hierarchically inclusive. Medical students benefit immensely from intergenerational learning relationships, not only with attending physicians but also with near-peer mentors such as residents and senior students. These relationships facilitate the safe transmission of tacit knowledge, help normalize uncertainty and vulnerability in clinical learning, and cultivate psychological safety, an essential foundation for honest dialogue and growth. Programs that intentionally structure such mentorship, including longitudinal clerkships or layered learning models, have been shown to enhance professional identity formation and foster a sense of belonging within the broader healthcare community.

**Table 1:** Proposed educational interventions from connection-centered viewpoint to cultivate relationally competent clinicians

Educational Interventions	Educational Strategy	Primary Purpose and Competency
Pre-Clinical Clerkship (early-year)	Lectures by various medical staffs	Shared mental models, role flexibility
	Internship at local healthcare and welfare facilities	Trust-building, sustained relationships
	Interdisciplinary discussion among faculties	Team-based problem solving, intellectual humility
	Problem-based learning on collaboration	Reflective learning, role understanding
Clinical Clerkship (senior-year)	Interprofessional rounds and case discussions	Team-based problem-solving, real-time collaboration
	Role-shadowing across professions	Perspective-taking, role understanding
	Vertical mentoring (residents, senior students)	Psychological safety, tacit knowledge transfer, identity formation
	Longitudinal clerkships	Trust-building, sustained relationships
	Interpersonal simulations	Empathy, negotiation, complex communication skills
	Interprofessional simulation scenarios	Shared mental models, role flexibility, real-world mirroring
	Narrative evaluations and 360-degree feedback	Reflective learning, relational performance feedback

At the same time, simulation-based education offers a powerful and flexible complement to real-world clinical experience, particularly when preparing students to navigate complex or emotionally charged interactions [36]. Traditional simulations have focused primarily on procedural skills or acute crisis management, but they should be expanded to include complex interpersonal scenarios that demand empathy, negotiation, and boundary-crossing collaboration. For example, simulations can replicate challenging conversations such as disclosing medical errors, coordinating end-of-life care with families and teams, managing conflict between departments, or reconciling ethical dilemmas involving multiple stakeholders [37]. These encounters offer a psychologically safe environment in which students can practice communication, receive feedback, and develop confidence before confronting similar challenges in actual patient care.

Interprofessional simulations, in particular, are uniquely positioned to dismantle rigid role conceptions and foster mutual accountability [38]. Joint training exercises involving students from medicine, nursing, pharmacy, and allied health programs can mirror the realities of emergency care, rehabilitation planning, or hospital discharge coordination. When such simulations are designed around shared goals, realistic complexity, and structured reflection, they help participants develop what educational theorists call shared mental models, a cornerstone of effective teamwork [39]. These experiences should be woven longitudinally into clinical education, rather than treated as isolated workshops, to normalize collaboration as an everyday practice.

Furthermore, educators must critically examine how institutional structures, assessment systems, and cultural norms either support or inhibit this relational approach [40]. Current evaluation systems continue to reward individual knowledge and task completion over collective sense-making, communication, and co-creation. To truly embed relational competence, assessments must evolve to capture how learners engage with others, how they reflect on interpersonal challenges, and how they contribute to collaborative care. Narrative evaluations, 360-degree feedback, and portfolio-based assessment tools aligned with real-world competencies can better represent the multidimensional nature of clinical performance.

The implementation of such a model demands faculty development, interinstitutional coordination, and a shift in institutional culture. Clinical educators must be trained not only in the clinical supervision of students, but in mentoring collaborative learning and modeling relational professionalism [41]. Academic leadership must prioritize time, space, and recognition for these pedagogical innovations, rather than marginalizing them as “soft” or supplemental. Indeed, labeling relational and collaborative competencies as “soft” skills can itself perpetuate institutional resistance and devalue their role in professional formation. Without such systemic support, clerkships risk becoming procedural exercises divorced from the collaborative reality of healthcare practice.

Ultimately, the purpose of clinical education is not merely to teach future clinicians what to do. It is to shape who they become and how they relate to others in a dynamic, interdependent healthcare world [42]. This transformation demands a redesign of clinical training that prioritizes connection, collaboration, and communication. By doing so, we can better prepare healthcare professionals who are not only technically capable, but also socially responsive, ethically grounded, and institutionally aware.

Educational innovation rooted in the essence of healthcare: toward practice-oriented medical education that cultivates multilayered collaboration

**Figure 2:**
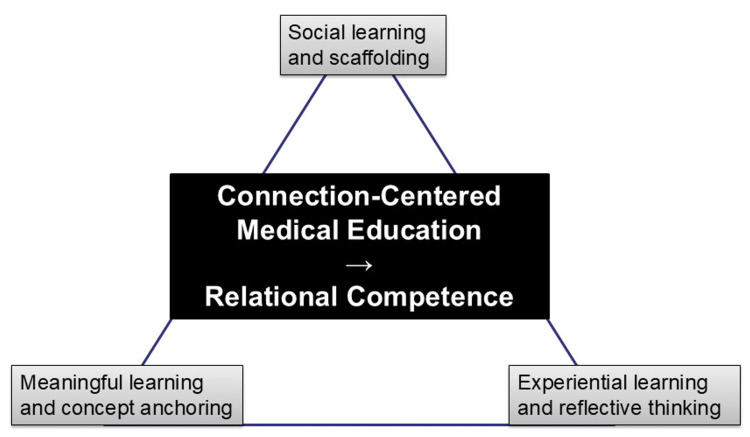
Educational theories underpinning connection-based learning.

In an era of rapid technological advancement, demographic change, and global health uncertainty, the defining quality of future healthcare professionals will not be individual technical brilliance alone, but their ability to build, sustain, and renew human connections [43]. Medical education must move beyond its traditional emphasis on individual excellence and instead be reimagined as a living network of relationships. This calls for a connection-centered curriculum that supports multi-layered, ethical, and enduring collaboration across all stages of training (Figure 2).

To realize this vision, the cultivation of connection must be deliberately and systematically embedded into the core architecture of the medical curriculum [44]. This requires moving beyond sporadic initiatives or elective modules and toward a comprehensive, vertically and horizontally integrated educational design that prioritizes relational competence as a foundational pedagogical principle, from preclinical education to postgraduate development and lifelong learning [45].

At the undergraduate level, this means redesigning basic science and early clinical exposure as collaborative platforms. Rather than treating anatomy, physiology, or pharmacology as isolated silos, curricula should promote integration across disciplines, dialogue among students, and structures for peer and near-peer mentorship. Drawing on Vygotsky’s Zone of Proximal Development (ZPD), junior learners benefit from guidance not only from faculty but also from slightly more advanced peers. This relational approach to learning reinforces both vertical structures and horizontal structures, aligning with the principles of relational pedagogy, which values learning as a socially embedded, co-constructed process [46]. This intergenerational support fosters mutual growth and creates relational continuity even within student cohorts. Longitudinal small group formats such as problem-based and team-based learning (PBL/TBL) should likewise aim to cultivate trust, shared responsibility, and collaborative negotiation.

In the clinical clerkship phase, relational learning must be embedded into daily activities. Clerkships should allow students to engage deeply in interprofessional and intraprofessional teams, experiencing healthcare as a collaborative process [47]. Evaluation tools such as multisource feedback and structured reflection should be used to assess not just what students know, but how they relate, adapt, and contribute within complex systems. Shulman’s concept of pedagogical content knowledge is particularly relevant here, as faculty must be trained not only in what to teach, but in how to teach relationally, modeling inclusive, system-aware professional behavior [48].

Postgraduate and continuing professional development must also prioritize relational competencies. Team leadership, conflict resolution, and intersectoral communication should become standard components of residency and fellowship programs. Simulation-based training, especially in high-pressure or ethically complex contexts, can help clinicians develop communication and collaboration skills essential for advanced care. These experiences, aligned with Kolb’s experiential learning cycle, foster reflective and ethically grounded practice [49,50].

Crucially, these educational transformations must be institutionally supported. Faculty development should prepare educators as facilitators of connection, capable of guiding relational dialogue, supporting diverse learners, and managing conflict across professional boundaries. Institutions must allocate resources and protected time for collaborative teaching, interprofessional education, and community-engaged learning. Accreditation and evaluation frameworks must evolve to recognize relational competencies as core to clinical excellence, on par with technical and cognitive performance.

Ultimately, connection-based education is not an optional enhancement; it is a necessary evolution. By embedding collaboration into every layer of training, medical education can produce professionals who are not only knowledgeable and skilled, but who also possess the relational and ethical capacities needed to lead, heal, and innovate in a profoundly interconnected world. Such professionals will be better equipped to navigate the moral ambiguities, systemic pressures, and emotional demands of modern healthcare. Their ability to sustain meaningful connections with patients, colleagues, and communities will be essential to both personal resilience and collective impact.

## Conclusions

In an era marked by systemic fragility, social fragmentation, and accelerating global health challenges, the future of medicine depends not solely on technical knowledge or individual excellence but on our capacity to connect. The most effective healthcare professionals will be those who can communicate across disciplines, collaborate across sectors, and build trust across generations and communities. This capacity for connection is no longer a desirable attribute; it is a core competency in the pursuit of sustainable, equitable, and ethical care. Medical education must rise to this reality. It must shift from an outdated model of isolated expertise to one that actively cultivates collaborative intelligence, the ability to learn with and from others, to navigate uncertainty together, and to co-create solutions in real time. This transformation requires more than curriculum reform; it calls for a redefinition of what it means to be a healthcare professional in a deeply interdependent world.

From preclinical integration and peer mentorship to interprofessional simulations, community engagement, and longitudinal clerkships, education must be designed as a relational journey. Learning environments must be structured to promote dialogue, mutual reflection, and shared purpose. Educators must be supported to model the relational competencies they seek to instill. Assessment systems must evolve to recognize the value of teamwork, communication, and inclusive problem-solving alongside clinical skill. Ultimately, the goal of medical education is not simply to prepare individuals to function within current systems but to empower them to transform those systems from within. If we can embed connection into the very fabric of training, we will cultivate professionals who do more than deliver care, they will heal the structures of care itself. In doing so, medical education can become a cornerstone of a more just, resilient, and connected healthcare future.
